# Miscellanies

**Published:** 1838-10-01

**Authors:** 


### MISCELLANIES.

### ANIMAL MAGNETISM.

IF the following report prove to be correct (and it is said to be authentic) we must, however humiliating it may be to our pride, withdraw all opposition to Mesmerism, and acknowledge ourselves to have been unreasonably sceptical on the occasion.

A young and hysterical female had suffered much from most painful menstruation, for some years. It was proposed to try the new agent to lull the pain of back and hypogastric region, during the first day of the dysmennorrhœa. For this purpose magnetized water was injected into the vagina, and a piece of Nickel applied to the loins. The effect was almost magical. The whole of the uterine system fell into profound Mesmeric coma, which lasted twelve hours, after which menstruation went on calmly and free from pain. The same process was employed at the next catamenial period, and with equal success. The third period passed without pain—and without any menstruation at all.

This was considered to be accidental, and that, at the fourth epoch, all would come right. The fourth passed, however, in the same way as the third, and the consequence of this obstruction was, morning sickness, and some qualms and caprices as to certain articles of diet. Soon after this, the mammæ enlarged a little, and the areola round the nipples became of a darker hue. Still later the young female became plumper about the abdomen than she used to be—and, finally, there was no doubt left as to the powers of the magnetized water. The MAGNATES MAGNETICI were now in rapture, and became satisfied that the advent of a young Mesmeric Shiloah was at hand, and that HE would exhibit the zoo-magnetic powers in their highest degree of perfection. They confidently predict that he will, not only have *clairvoyance* at his *finger's ends*, but that he will be able to magnetize and somnolize every living creature at the distance of the antipodes, if desirable. As our antipodes are in Australia, let the kangaroos look out sharp, or they will be "*caught napping*," and find their way into our zoo-logical gardens.

The heads of the MAGNATES are nearly turned by this event, as they prognosticate, and not without reason, that a new race of mortals—perhaps immortals—is about to appear—something of the Minerva breed—struck out of Being's brains—from brains struck out of Beings. And as successive revolutions of our earth prepared the way for successive races of animals, so the Magnates Magnetici are probably destined to introduce a new code of laws into the physical, or even moral world —the old laws of Nature and Nature's God beginning now to be effete.*

But let the philosophers of the new light ponder a little on the results of their discoveries. When the modern Prometheus succeeded in infusing a spark of life into the man-monster at Geneva, he little thought that he was bringing into the world a demon over whom he would have no control; but who, on the contrary, would embitter the days of his creator, and destroy the lives of innocent victims! So the magnetizing Frankensteins may be unchaining a monster who may overwhelm the liberators in ruin or destruction.

Let us come a little closer to the question. Animal magnetism must either be true or false—a fact or a fiction. Suppose it be true:—and see the consequences. By a single wave of the hand, we deprive a female of all sense, and throw her into such a profound sleep that the teeth may be pulled out of her head, without the slightest consciousness on her part. Should such a power on the one side, and such susceptibility on the other, be once established, no female in the realm, however high or low her station, would be one day safe from the machinations of the wicked and licentious! In short, the whole foundations of society would be broken up, and every fence of virtue and honour would be levelled in the dust!

Fortunately for society, animal magnetism is a fiction—a falsehood. But even in this point of view, is it harmless because it is baseless? By no means. In the first place, it has, even already, affixed a stigma on the medical profession, whose members were considered to be too intelligent and too enlightened, to become the dupes of impostors, and to give credence to absurdities which would scarcely be listened to in the wilds of America or the interior of Australia. Medical men were generally supposed to know something of anatomy and physiology, and to be amongst the foremost to bear testimony to the infinite wisdom displayed in adapting every organ of the human body to its proper and peculiar function. But now the public is astonished to learn that some of the magnates of the profession have repudiated this divine adaptation, and proclaimed the astounding fact that the finger, or the epigastrium can perform the functions of the eye, thus proving that the Architect of man was a bungler and went to great and unnecessary trouble in constructing complicated apparatuses for specific functions, when any, even the most superficial part of the body could, by the mandate of man himself, perform these functions allotted by the Deity to their proper organs. But this is not all. The medical profession and the world at large saw clearly, and freely acknowledged that man had no *prescience*—knew nothing, in fact, but what he gathered from his physical senses—except where there was a special revelation from Heaven; yet now there are medical men who implicitly believe that a flatulent, hysterical, and impudent baggage can *prophecy* —can see events that are in the womb of fate—and predict the operation of medicines of which she is totally ignorant!! What must the world think of believers in such blasphemous mummeries? We pity and despise the poor Hindoo who worships his cow, and the Chinese who flogs his Josse-God, when he is not favoured with a fair wind in his junk; but these benighted people were born and brought up in the darkness of idolatry—an excuse which medical men cannot plead.

We shall allude to only one more consequence of animal magnetism, contemplated as a delusion. It ena-

* It is expected that a celebrated anatomist will stand "foster-father" on this occasion, and will baptize the magnetic infant in the Greek ritual, and under the name of St. Nickel-Ass.

bles every artful wench, under the manipulations of the magnates, or even of their brainless disciples, to set up as a prophetess, and, in her Mesmeric ravings, not only to see but *reveal* the transactions that are going on amongst her neighbours. Every person whose brains are not in a state of magnetic coma, must see, at a glance, what irreparable mischief a PYTHIA of the modern Delphi may produce, by giving her tongue liberty to wag, and her jealousies or hatreds ample scope to victimize at the expense of others. It is said of the original Pythia that, when Greece began to lose its independence, means were found to corrupt the Pythia —and Demosthenes complains that, in his time, "she spoke as Philip of Macedon would have her." The public will readily perceive the applicability of this passage to certain Macedonian monarchs who are working the tripod at present, with little credit to themselves and much scandal to the profession at large. Extremes approximate. Strange that philosophers so far elevated above the common credulity of the world, should yet be so credulous themselves, as to believe in the supernatural powers and prophetic visions of ignorant, vulgar, and mendacious impostors!!

These observations are not levelled at, nor intended for, any individual, but they are addressed to every one who sanctions by his presence, or supports by his pen, one of the most barefaced and outrageous impostures that was ever foisted on the public, or that disgraced the members of a liberal and enlightened faculty.

---

After the above observations had been penned, we met with the following most extraordinary passage in the Medical Gazette, by Mr. Mayo.

"Hitherto the tendency of physiological science has inclined towards materialism. Every new discovery regarding the nervous system up to the present time has tended only to shew more and more the dependence of the mind on the bodily organization, by parcelling out and assigning separate mental operations to separate parts of the brain or nerves. And, however, by reasoning drawn from other sources, one has shaken off or struggled against the weight of the physiological argument, one has always felt its influence in straitening those chinks and openings, through which one has caught glimpses of *spiritual existence*, and its force in habituating us to attribute more and more influence and compulsion to matter.

But the phenomena which I now mention that I have witnessed, and which are already admitted as part of Nature by some of the first surgeons and physicians of France and Germany, seem directly to lead to other conclusions, and to support those convictions which we derive from a higher philosophy.

I think that the phenomena of *prevision and transposition of sensation* (and those of clairvoyance likewise, if the latter are true, and there is any thing in them beyond the workings of an over-active imagination) naturally lead to the supposition, that they result from the workings of a *spiritual nature, in a certain independence of those bodily organs to which it is normally closely tied and bound.* It is conceiveable, that, in such cases as I have described, when all the common avenues of sensation are concluded—when eye-sight, taste, touch, hearing, are suspended—*and when a sort of vision is sensibly exercised by some part of the common surface of the body*—that these phenomena arise from *the mind being in part dislocated and displaced from her corporeal tenement, holding on with misplaced attributes to unaccustomed points and corners of the frame.* It is conceivable, again, that in that wrapt and mysterious state, in which the individual is giving utterance to remote anticipations that are *strangely verified, the mind is acting independently of its usual organs, and, with the character of spirituality, is freed from the restraints of time, as in clairvoyance (if that state ever exist), it would appear to be partially free from the restraints of space.** Man, we are told, was made in the image of God: these may be partial revealings of the parity of the

---

* Med. Gazette, Aug. 11, 1838.

spiritual nature of the created being to that of his Creator. *There are many things in tradition and popular belief, which would lead one towards such a hypothesis as that which I have advanced,* as they might equally find their solution and explanation in it.

From Homer to Shakspeare and Scott, the great observers of mankind and knowers of human nature, have represented the prophetic spirit as occasionally manifesting itself immediately before the approach of death, when it may be supposed that the soul is loosening herself, from her corporeal residence. And who is there, who has not himself met with perfectly authenticated instances of communication strangely felt, rather than made, of the time of the dissolution of an absent friend? Perhaps in the origin of such superstitions (for superstitions we habitually consider them, although from their universality they seem to deserve the consideration even of philosophers) there may be something in common with the source of the wonders of Mesmerism.

HERBERT MAYO.

August 8, 1838."

Upon such an explanation or *apology* for clair-voyance and transposition of the senses, we dare not trust ourselves with any remarks. We defy Germany itself to produce such a specimen of transcendental psycho-physiology as the above. This is psychology coming to the rescue of animal-magnetism with a vengeance! What does Dr. Elliotson say to this doctrine about the "*soul*?"

P.S.—A grand explosion of animal magnetism has taken place, and, on the 9th of August, Mr. Wakley has blown the *disjecta membra* of Mesmerism into the air, leaving scarcely a wreck behind. Mr. Wakley owed this to the profession; for the publication of the reports in the Lancet tended very much to keep up the delusion—especially as the magnates evidently calculated on the support of that periodical, seeing that there was somewhat of a leaning towards Mesmerism in the reporter, if not in the editor. But the magnates reckoned without their host, and in changing King Log, the reporter in the North London Hospital, for King-Stork, the consumer, in Bedford Square, they made a woeful mistake! It was not likely, indeed, that he who made Mons. Chabert eat up his own words—a much more ungrateful task than eating his own fire—should allow another Monsieur to play his antics long in Wigmore-street. As the detection of the impostures is now before the public in the Lancet, we need only quote Mr. Wakley's final conclusion. "After the girls had departed, Mr. Wakley made a few remarks on what had been witnessed, and declared that, in his opinion, the effects which were said to arise from what had been denominated 'animal magnetism,' constituted one of the completest delusions that the human mind ever entertained." Every person present, except the Baron du Potet, and Dr. Elliotson, concurred in this sentiment.

We may now consider that a fatal and final blow has been given to "animal magnetism," and we envy not the feelings of those who were credulous enough to lend an ear to such jongleries. What new Greek name will Mr. Mayo *now* search out for the mummery of Mesmerism? We can tell him that he need not take the trouble of hunting through his Lexicon. He need not even consult "Johnson." The Slang Dictionary, under the head of "HUMBUG" will supply him with a very proper, though not very erudite term, for the new quackery; or, if he prefers a more classical appellation, let him christen the new science by the title of NICKEL-ASSERY. Mr. M. thinks he has left "EXONEURISM" in a better condition than that in which he found it. We are of a very different opinion, and suspect that his extravaganzas have contributed not a little to hasten its downfall. We shrewdly suspect, too, that Dupotet and Elliotson entertain a similar conviction, and that these gentlemen secretly curse the hour when Mr. Mayo "lent his fostering offices towards Mesmerism." Many a time will they groan in spirit—

"Non tali auxilio, nec defensoribus istis

MESMER eget.*

It is worthy of notice, that not one of the seven medical journals published in Great Britain has supported "animal magnetism." Four of them have now repudiated it, and three have preserved silence. It needs not a prophet to foresee that if the three journals in question speak out, it will be on the wrong side for Mesmerism.

It will hardly be believed, but we have authentic proofs of the fact, that even after this exposure in Bedford Square, a private somnambulary is still kept established in the West End, under the highest patronage and superintendance, where *gulls* from the country are *clawed* four times a week for a sovereign, viz. five shillings per *Seance!!*

---

RELATIONSHIP BETWEEN EPILEPSY AND APOPLEXY.

A gentleman in the legal profession, aged 58, who held an official situation where great mental labour was required, became affected with epileptic, or epileptiform fits, which usually came on early in the morning, before leaving bed, and exhibited convulsive struggles, generally with loss of consciousness for a short time, followed by sleep and lassitude. The next day he was usually fit for his mental labours, and experienced no deterioration of his intellectual powers. The attacks became gradually more frequent, and for some time, returned pretty nearly at intervals of a fortnight, four o'clock in the morning being the hour of invasion. No medical man witnessed the attack, in consequence of the period of its occurrence. But the wife of the patient described these attacks accurately, and no other inference could be drawn but that they were epileptic. They had continued more than a year when the reporter saw him. There were no evidences of any cerebral lesion besides the fits above mentioned. He complained a little of his memory; but at 58 years of age, the memory begins to fail in most people. The digestive organs were attended to, counter-irritation to the nape of the neck, and small doses of the nitrate of silver were given for a few weeks; but as the attacks increased in frequency, rather than diminished, the nitrate was discontinued, and only ordinary aperients employed, with light diet, and temperance in drink. On Monday morning, the 21st May, at the usual hour—4 o'clock—he felt his usual attack coming on; but, alas! it was his last one. Instead of epilepsy, it proved to be decided apoplexy, which terminated life, in about 36 hours, despite of active treatment.

On dissection, the ventricles were found distended by a considerable quantity of clear water, evidently of long standing. The vascular system of the brain, but especially the venous, was remarkably congested throughout, but there was no rupture of vessels or effusion of blood. The brain itself, however, particularly about the crura cerebri, was in a state of ramollissement, so as to be incapable of dissection. This was still more especially the case in regard to the cerebellum, which was almost in a state of liquefaction, and would not bear handling.

Now it is not a little extraordinary that, with such a state of brain, this individual was able to use his intellectual powers, with scarcely any diminution, two or three days before his death! This puzzles our physiology and pathology. The case is also interesting, as shewing how easily epilepsy may merge into apoplexy, and prove fatal.

---

* The admirable tact with which Mr. Wakley contrived to "puzzle the conjurors," (including those notable performers, the Okey's), by substituting lead for nickel, and nickel for lead, has drawn forth the following jeu-d'esprit:—"Why did Wakley change O'Key's name from the feminine to the masculine gender, when he cheated her out of the miraculous metal? Because he then made her *Nickel-O'Minus.*"

